# Perspectives in Training and Professional Practice of Cardiac Surgery in Latin America

**DOI:** 10.21470/1678-9741-2022-0125

**Published:** 2023

**Authors:** Mateo Marin-Cuartas, Dominique Vervoort, Juan Roberto Contreras, Ovidio A. Garcia-Villareal, Alejandro Escobar, Javier Ferrari, Eduard Quintana, Rafael Sadaba, Carlos A. Mestres, Victorio C. Carosella, Rui M. S. Almeida, Victor Dayan, Latin American Association of Cardiac and Endovascular Surgery (LACES)

**Affiliations:** 1 University Department of Cardiac Surgery, Leipzig Heart Center, Leipzig, Germany.; 2 Division of Cardiac Surgery, University of Toronto, Toronto, Ontario, Canada.; 3 Surgery Department, Universidad de la Frontera, Temuco, Chile.; 4 Mexican College of Cardiovascular and Thoracic Surgery, Mexico City, Mexico.; 5 Universidad CES, Medellin, Colombia.; 6 Colegio Argentino de Cirujanos Cardiovasculares, Buenos Aires, Argentina.; 7 Department of Cardiovascular Surgery, Hospital Clínic de Barcelona, Barcelona, Spain.; 8 Department of Cardiac Surgery, Complejo Hospitalario de Navarra, Navarra, Spain.; 9 Department of Cardiovascular Surgery, University Hospital Zürich, Zürich, Switzerland.; 10 Department of Cardiothoracic Surgery, University of the Free State, Bloemfontein, South Africa.; 11 Instituto Cardiovascular San Isidro, Sanatorio Las Lomas, Buenos Aires, Argentina.; 12 Centro Universitário Fundação Assis Gurgacz, Cascavel, Paraná, Brazil.; 13 Centro Cardiovascular Universitario, Montevideo, Uruguay

**Keywords:** Education, Cardiac Surgery, Latin America, Professional Practice, Mentoring, Leadership, Surgeons

## Abstract

**Introduction:**

There is a lack of information about cardiac surgery training and professional practice in Latin American (LATAM) countries. This study is the first comparative analysis of cardiac surgical training and professional practice across LATAM and provides the fundamentals for future academic projects of the Latin American Association of Cardiac and Endovascular Surgery (LACES).

**Methods:**

International survey-based comparative analysis of the training and professional practice of cardiac surgeons across LATAM. Trainees (residents/fellows) and staf (graduated) surgeons from LATAM countries were included.

**Results:**

A total of 289 respondents (staf surgeons N=221 [76.5%]; residents/fellows N=68 [23.5%]) from 18 different countries participated in the survey. Most surgeons (N=92 [45.3%]) reported being unsatisfied with their salaries. Most respondents (N=181 [62.6%]) stated that it was difficult to obtain a leadership position, and 149 (73.8%) stated that it was difficult to find a job after completing training. Only half of the trainee respondents (N=32 [47.1%]) reported that their program had all resident spots occupied. Only 22.1% (N=15) of residents/fellows were satisfied with their training programs. The majority (N=205 [70.9%]) of respondents would choose cardiac surgery as their specialty again. Most surgeons (N=129 [63.9%]) and residents/fellows (N=52 [76.5%]) indicated that the establishment of a LATAM cardiac surgery board examination would be beneficial.

**Conclusion:**

Modernization and standardization of training, as well as greater access to opportunities, may be required in LATAM to increase professional satisfaction of cardiac surgeons and to reduce disparities in the specialty. Such changes may enhance the regional response to the dynamic challenges in the feld.

## INTRODUCTION

Training in cardiac surgery varies substantially across the world^[1]^. These differences are not only evident globally, but also in a regional context^[2]^. Cardiac surgery training and professional practice difer significantly across Latin American (LATAM) countries. First, there is discordance on how the specialty is named across the continent, using the terms cardiac, cardiothoracic, and cardiovascular surgery interchangeably. Second, the content and duration of the training considerably vary from country to country and even within countries, and the legally attributed roles to the specialty are not homogenous across LATAM countries. Moreover, salaries, workload, surgical volumes, job opportunities, and leadership positions significantly vary within countries and even more across the continent. Finally, there is a lack of officially reported and collected data in LATAM, and therefore, the current professional situation is unknown.

Cardiac surgery is a dynamic and continually evolving specialty. Thus, the challenges that cardiac surgeons must deal with are complex and transcend borders^[3]^. Hence, constant self-evaluation, improvement, and creative solutions are required to solve these problems. Moreover, LATAM countries have an additional hurdle: the lack of economic resources and limited access to novel technologies. Therefore, the unification of eforts is necessary to recognize the weaknesses and strengths of LATAM as well as to identify our current situation as a continent, thus helping to define a starting point for a common continental improvement pathway.

In response to that imminent need, the Latin American Association of Cardiac and Endovascular Surgery (LACES) works hard to recognize and address the abovementioned disparities and aims to unify and standardize cardiac surgery training programs in LATAM. The current project analyzes the cardiac surgical landscape in LATAM, aiming to have an actual and contemporary overview of the training and professional situation among LATAM cardiac surgeons. This study is a descriptive analysis of 1) cardiac surgical training and 2) professional practice after cardiac surgical training in LATAM countries.

## METHODS

### Study Design

**Fig. 1:**
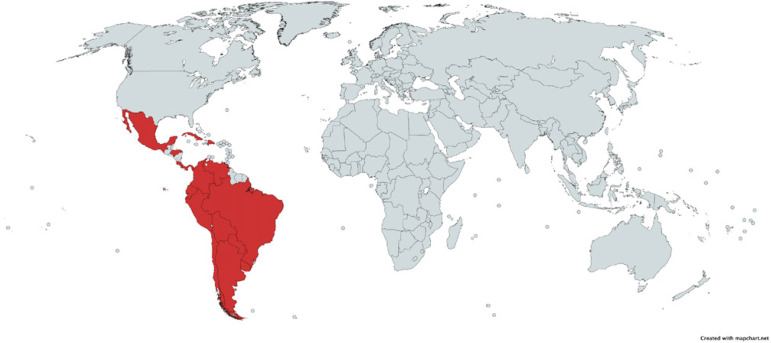
Countries of origin of eligible survey respondents.

We performed a descriptive cross-sectional study. Residents and cardiac surgeons were contacted by email from the LACES database and invited to answer a voluntary survey. Information was anonymous. The survey was conducted in English. Given the small number of surgeons in some countries, demographic information, such as age, could only be answered in the survey as ranges (*e.g.*, 40-50 years), but not with an exact value, thus ensuring the data anonymity. Hence, exact median or mean values cannot be calculated. The questionnaire was constructed considering the following domains: training, research and academic participation, professional satisfaction, financial retribution, and workload. Questions to address each of these domains were constructed. A pilot study was performed among random surgeons and residents to ensure the questionnaire’s comprehensiveness and obtain feedback regarding additional or irrelevant questions. Reliability was assessed based on internal consistency evaluation. The survey was divided into three parts: 1) general questions for all participants, 2) questions only for trainees (residents/fellows), and 3) questions only for staf (graduated) surgeons. The survey can be seen in the Supplementary Appendix. Answers were collected from March to April 2021. Participants from the following countries answered the survey (Figure 1): Argentina, Aruba, Bolivia, Brazil, Chile, Colombia, Costa Rica, Cuba, Dominican Republic, Ecuador, Guatemala, Honduras, Mexico, Panama, Paraguay, Peru, Uruguay, and Venezuela.

### Statistical Analysis

The normality of data was assessed using the Shapiro–Wilk test. Normally distributed data were presented as mean ± standard deviation. Non-normally distributed data were presented as median with interquartile ranges. Non-parametric variables were assessed and compared using Student’s *t*-test or chi-squared test, as appropriate. All data were tabulated, and analyses were performed using Microsoft® Excel® (Microsoft, Redmond, Washington, United States of America) and Prism™ (GraphPad LLC, San Diego, California, United States of America). Statistical significance was accepted at *P*<0.05. To avoid violating confdentiality and ensure the anonymity of the respondents given the small number of surgeons in some participating countries, some results (*e.g.*, age) are limited to ranges rather than expressed as specific numeric values.

## RESULTS

### Respondents’ Demographics

Thirty-seven responses were excluded based on country. After excluding ineligible responses, analyses were performed on the remaining (N=289) responses. Respondents originated from 18 different countries (Figure 1) with a median age between 40-50 years. Respondents’ demographics are summarized in Table 1.

**Table 1:** Demographics of survey respondents.

		Total (N=289)	Surgeons (N=221)	Residents/Fellows (N=68)	*P*-value
**Sex**	Female	36 (12.4%)	21 (9.5%)	15 (23.7%)	0.011
	Male	252 (87.2%)	199 (90.0%)	53 (76.3%)	
	Prefer not to say	1 (0.3%)	1 (0.5%)	0 (0%)	
**Age (years)**	Under 30	43 (14.9%)	2 (0.9%)	41 (47.7%)	< 0.001
	30 – 40	103 (35.6%)	58 (26.2%)	45 (52.3%)	
	40 – 50	73 (25.3%)	73 (33.0%)	0 (0.0%)	
	50 – 60	62 (21.5%)	62 (28.1%)	0 (0%)	
	60 – 70	20 (6.9%)	20 (9.0%)	0 (0%)	
	Over 70	6 (2.1%)	6 (2.7%)	0 (0%)	
**Race/Ethnicity**	White	195 (67.5%)	151 (68.3%)	44 (64.7%)	0.322
	Mestizo	86 (29.8%)	65 (29.4%)	21 (30.9%)	
	African American	1 (0.3%)	0 (0.0%)	1 (1.5%)	
	Other	7 (2.4%)	5 (2.3%)	2 (2.9%)	
**Leadership position**	Chair/Head	81 (28.0%)	81 (34.8%)	0 (0%)	< 0.001
	Consultant	71 (24.6%)	71 (30.5%)	0 (0%)	
	Chief resident	13 (4.5%)	0 (0%)	13 (19.1%)	
	Societal leadership	17 (5.9%)	17 (7.3%)	0 (0.0%)	
**Hours of work per week**	Less than 40	26 (9.0%)	25 (11.3%)	1 (1.5%)	< 0.001
	40 – 80	175 (60.6%)	148 (67.0%)	27 (39.7%)	
	80 – 120	71 (24.6%)	37 (16.7%)	34 (50.0%)	
	More than 120	17 (5.9%)	11 (5.0%)	6 (8.8%)	
**Institution**	Total responded	N=270	N=202	N=68	0.119
	Public	156 (57.8%)	111 (55.0%)	45 (66.2%)	
	Private	114 (42.2%)	91 (45.0%)	23 (33.8%)	
**Academic degrees**	Total responded	N=270	N=202	N=68	< 0.001
	Master’s only	44 (16.3%)	38 (18.8%)	6 (8.8%)	
	PhD only	43 (15.9%)	40 (19.8%)	3 (4.4%)	
	Both	7 (2.6%)	7 (3.5%)	0 (0.0%)	
	Neither	176 (65.2%)	117 (57.9%)	59 (86.8%)	
**Years of experience**	Total responded	N/A	N=202	N/A	N/A
	Less than 5 years		38 (18.8%)		
	5 – 10 years		34 (16.8%)		
	11 – 15 years		32 (15.8%)		
	16 – 20 years		32 (15.8%)		
	More than 20 years		66 (32.7%)		
**Current year of cardiac surgery training**	Total responded	N/A	N/A	N=67	N/A
	1			15 (22.4%)	
	2			12 (17.9%)	
	3			16 (23.9%)	
	4			17 (25.4%)	
	5			2 (3.0%)	
	6+			5 (7.5%)	

N/A=not applicable

### Work and Leadership

Most respondents reported working 40-80 hours (N=175, 60.6%) or 80-120 hours (N=71, 24.6%) per week. Few reported working < 40 hours per week (N=26, 9.0%). Weekly working hours were significantly higher for residents and fellows compared to surgeons (*P*<0.001).

The majority (N=205, 70.9%) of respondents reported choosing cardiac surgery as their specialty of training again if given the option, whereas 23.5% (N=68) were unsure, and 5.5% (N=16) would prefer a different specialty. Among surgeons, 72.4% (N=160) would choose cardiac surgery again, whereas 5.9% (N=13) would not. Among residents and fellows, 66.2% (N=45) reported choosing cardiac surgery again, whereas 4.4% (N=3) would not.

Forty-one (14.2%) respondents stated that leadership positions are widely available and accessible in their institution, city, and/or country. In contrast, 62.6% (N=181) respondents stated that it was difficult to obtain a leadership position in some capacity. The remaining respondents did not consider leadership positions, either due to lack of interest (N=28, 9.7%), lack of time (N=6, 2.1%), or having to take up too many responsibilities with little or no pay (N=27, 9.3%).

### Surgeon-Specific Responses

A total of 221 surgeons (76.5%) responded, of whom 9.5% (N=21) were female and 90.0% (N=199) were male. Respondents stemmed from 16 different countries with a median age between 40-50 years. Among the surgeons that responded, 91.4% (N=202) completed surgeon-specific questions.

**Fig. 2:**
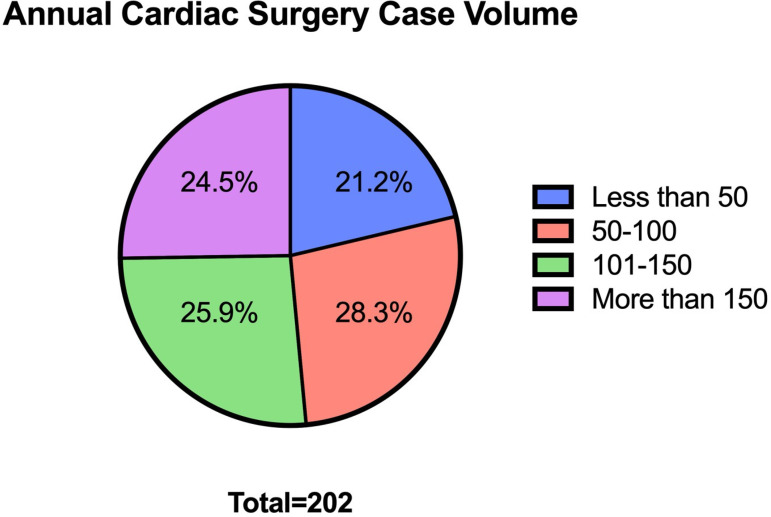
Annual cardiac surgical case volume by staf (graduated) surgeon respondents (N=202).

Most surgeons worked in public centers (N=111, 55.0% *vs.* N=91, 45.0% in private centers) and completed fellowship training (N=147, 65.1% *vs.* N=74, 34.9%). The most common fellowships pursued were congenital heart surgery (N=40, 18.9%), heart transplantation (N=18, 8.5%), and aortic surgery (N=17, 8.0%). Transcatheter interventions fellowships and minimally invasive cardiac surgery fellowships were completed by 18 (8.5%) and 15 (7.1%) respondents, respectively. Annual cardiac surgery case volumes were evenly distributed (Figure 2). Two-thirds of surgeons trained residents (N=139, 68.8%), and nearly three-quarters of them (N=144, 71.3%) performed research to some extent.

Surgeons’ training ranged from less than four years (N=51, 25.2%) to more than eight years (N=15, 7.4%), with a median of 5-6 years (Table 2). Also, 18.8% (N=38) reported not having undergone general surgery training, while most conducted either 1-2 years (N=65, 32.2%) or 2-4 years of general surgery training (N=68, 33.7%), with a median of 1-2 years. The median cardiac surgery training surgeons underwent was 3-4 years, ranging from 1-2 years (N=8, 4.0%) to more than four years (N=88, 44.2%). Critical care training varied widely from having received no dedicated training (N=56, 27.8%) to more than two years of training (N=62, 30.8%), with a median of 3-6 months. Most of surgeons (N=129, 63.9%) indicated that establishing a regional cardiac surgery board exam would be beneficial, while 5.9% (N=12) were completely against it. Three-quarters of surgeons (N=149, 73.8%) stated that it was difficult to find a cardiac surgery job after completing training.

**Table 2:** Training durations reported by cardiac surgeons, residents, and fellows.

		Surgeons (N=202)	Residents/Fellows (N=68)	*P*-value
**Total length of training**				0.300
	Less than 4 years	51 (25.2%)	11 (16.2%)	
	5-6 years	93 (46.0%)	38 (55.9%)	
	7-8 years	43 (21.3%)	12 (17.6%)	
	More than 8 years	15 (7.4%)	7 (10.3%)	
**Length of general surgery training**				< 0.001
	None	38 (18.9%)	26 (38.2%)	
	Less than 1 year	17 (8.4%)	9 (13.2%)	
	1-2 years	65 (32.2%)	11 (16.2%)	
	2-4 years	68 (33.7%)	12 (17.6%)	
	More than 4 years	14 (6.9%)	10 (14.7%)	
**Length of cardiac surgery training**				0.010
	Less than 1 year	0 (0.0%)	3 (4.4%)	
	1-2 years	8 (4.0%)	2 (2.9%)	
	2-4 years	103 (51.8%)	27 (39.7%)	
	More than 4 years	88 (44.2%)	36 (52.9%)	
**Length of critical care medicine training**				0.006
	None	56 (27.8%)	27 (39.7%)	
	Less than 3 months	25 (12.4%)	17 (25.0%)	
	3-6 months	25 (12.4%)	6 (8.8%)	
	6-12 months	9 (4.4%)	3 (4.4%)	
	1-2 years	24 (11.9%)	8 (11.8%)	
	More than 2 years	62 (30.8%)	7 (10.3%)	

Surgeons’ monthly salaries widely varied, with a median between 2,500-5,000 USD (or United States Dollar). Most surgeons reported being unsatisfied (N=92, 45.3%) or moderately satisfied (N=86, 42.4%) with their salary. Most surgeons (N=165, 82.1%) reported having received a salary during their residency training. Nearly one in fve surgeons (N=41, 20.4%) stated they had to pay for residency training, of whom 73.2% (N=30) had to take loans to aford to pay for residency.

### Resident/Fellow-Specific Responses

Sixty-eight residents and fellows (23.5%) responded (23.7%, N=15 female *vs.* 76.3%, N=53 male). Respondents had 14 different countries of origin with a median age between 30-40 years. All residents and fellows completed all or most resident/fellow-specific questions.

The anticipated median duration of training for residents and fellows is expected to be 5-6 years. Residents and fellows completed a median of less than one year of general surgery training and less than three months of critical care medicine training and are expected to complete a median of more than four years of cardiac surgery training (Table 2). Most residents and fellows received a salary during training (N=58, 85.3%). One in fve (N=13, 19.1%) reported having to pay for residency training.

**Fig. 3:**
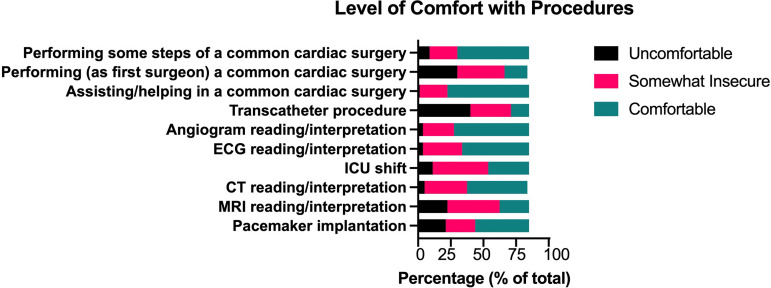
Residents’ and fellows’ level of comfort in performing procedures. CT=computed tomography; ECG=electrocardiogram; ICU=intensive care unit; MRI=magnetic resonance imaging.

The comfort levels of residents and fellows in different clinical and surgical tasks varied considerably (Figure 3). Most (N=41, 60.3%) reported independently performing less than one case per week, on average, whereas contributions to essential parts of operations vary (N=21, 30.9% less than one per week; N=13, 19.1% one per week; N=22, 32.4% 2-5 per week; N=12, 17.6% more than fve per week). A third (N=23, 33.8%) of residents and fellows anticipated having performed less than ten procedures independently by the end of their training compared with 30.9% (N=21) expecting to have performed more than 50 independent procedures. Half of the respondents (N=32, 47.1%) reported that their program had all resident spots filled, whereas 11.8% (N=8) had one open spot, 17.6% (N=12) had two open spots, and 23.5% (N=16) had more than two resident spots unfilled. A median of 2-4 night shifts were performed per week, with 8.8% (N=6) reporting 5-7 night shifts per week. Satisfaction with training programs varied, with 22.1% (N=15) being satisfied, 48.5% (N=33) moderately satisfied, and 29.4% (N=20) unsatisfied.

Most respondents (N=61, 89.7%) stated they wish to pursue or are currently pursuing fellowship training. The most reported fellowships being pursued or expected to pursue included minimally invasive cardiac surgery (N=18, 26.5%), aortic surgery (N=11, 16.2%), congenital heart surgery (N=12, 17.6%), and transcatheter interventions (N=10, 14.7%). Most of residents and fellows (N=52, 76.5%) were in favor of the development of a regional cardiac surgery board exam, whereas 2.9% (N=2) were opposed. Research was commonly but variably performed during training (N=48, 70.6%).

## DISCUSSION

The current study represents the first analysis of the current training and professional practice situation in cardiac surgery in LATAM countries. Knowing this information is essential to accelerate continuous improvement and standardize cardiac surgical training and practice in LATAM. It also helps to improve the professional satisfaction of LATAM cardiac surgeons and reduces disparities in the specialty across the continent. The main findings of this study are summarized in Table 3.

**Table 3:** Main survey findings.

Positive aspects	Negative aspects
Most cardiac surgeons would choose training in cardiac surgery again.	There is a significant gender and racial disparity among cardiac surgeons in LATAM.
A significant number of surgeons completed a clinical fellowship after finishing their initial cardiac surgical training. Likewise, most residents plan to pursue a fellowship once they finish their training.	According to the respondents, there is a lack of job opportunities and leadership positions in LATAM. Moreover, most surgeons were not satisfied with their salaries.
There is a general agreement on the benefts of a LATAM cardiac surgery board exam, similar to those performed by the European Board of Cardiothoracic Surgery and the American Board of Thoracic Surgery.	Despite good clinical training, academic degrees such as a master’s degree or PhD are uncommon amongst the respondents.
There is a high interest of both surgeons and trainees in research activities.	Only one-third of residents/fellows are satisfied with their training programs.
	According to the respondents, only half of the training spots in cardiac surgery are occupied.

LATAM=Latin-American

Although there is an overall feeling of satisfaction within the specialty, cardiac surgeons in LATAM report difficulties finding job opportunities and are unsatisfied with their salaries. There is a false feeling of “competition” for job opportunities in large cities and a general belief that there is an “over-ofering” of surgical training programs in LATAM. However, LATAM is still underperforming cardiac surgical volumes due to an insufficient workforce^[4]^. Moreover, according to the survey, only around 50% of the available training spots in LATAM are currently occupied. Hence, if the conditions are improved in smaller towns/centers, accurate geographical distribution of LATAM surgeons could be achieved^[5]^.

To improve the satisfaction with salaries, the implementation of regional “tarif systems”, like in Germany^[6]^, could regulate the wages. The tarif system establishes the minimally legally accepted salary for trainees and surgeons according to their years of experience. Most respondents stated that it is difficult to obtain a leadership position in LATAM. However, most respondents with a leading position also have academic titles such as a master’s degree and PhD and/or completed advanced training fellowships. In other countries outside of LATAM^[7]^, surgeons obtaining leadership positions usually have the most complete surgical and academic profiles. It is the responsibility of associations such as LACES to ofer grants and broaden the global network to allow their members access to academic, research, and advanced surgical training programs. This would significantly improve LATAM surgeons’ professional profile and increase the opportunities when applying to leadership positions besides considerably increasing the quality of cardiac surgery in LATAM.

The number of weekly work hours for residents in LATAM is comparable to the number of the United States of America^[8,9]^, but it is considerably higher than in most European countries^[10,11,12]^. However, except for Brazilian trainees^[13]^, according to the survey, LATAM residents per form significantly less own “skin-to-skin” cases during their whole training in comparison with European and North American training programs^[7,10]^. This evident disbalance between surgical learning and work hours must be corrected in LATAM. Moreover, standardized “logbooks” for competence-based learning, where procedures can be rigorously documented, might significantly improve the training^[3]^. Competency-based training and evaluation ofer an option to compensate for inequality among trainees due to patient volume differences in training centers. The opportunities provided by simulation to rehearse procedures and management of challenging situations before encountering them in real life cannot be overemphasized. Diferent countries have published multiple successful experiences: the United States of America and Canada ofer simulation boot camps to learn how to conduct basic skills^[14]^. The Brazilian Society of Cardiovascular Surgery frequently hosts wet lab courses for trainees^[13]^. The national cardiac surgery associations in the United Kingdom and Germany ofer a broad curriculum of courses covering operative and nonoperative skills^[15]^. However, surgical simulation is expensive, and access to good quality simulators is, therefore, limited. Hence, LATAM training programs need to find sufficient funding to implement a formalized longitudinal simulation curriculum to improve the training of a new generation of cardiac surgeons.

Cardiac surgery is going through the so-called endovascular revolution, and surgeons need nowadays to obtain transcatheter expertise^[16,17]^. However, only a minority of respondents completed advanced fellowship training in minimally invasive surgery and transcatheter interventions. Hence, training programs must respond to the imminent need to update their contents and competencies. In addition, compared to more senior surgeons, younger generations of cardiac surgeons in LATAM have less exposure to critical care and general surgery training. Therefore, including a more comprehensive critical care and basic general surgery training in the current cardiac surgery pensums might significantly improve the training’s quality as well as increase the confidence of younger surgeons in the management of perioperative complications.

A particular focus in research is of utmost importance to increase innovation in the cardiac surgery feld. LATAM countries have plenty of socioeconomic problems, and, therefore, innovation is more than needed to face some of our issues in the treatment of cardiovascular diseases. This is how national and international scientific associations must join forces to increase funding of research projects in LATAM to encourage surgeons to increase research and innovation “made in LATAM”. Moreover, a LATAM-wide database is required to facilitate the performance of clinical trials and improve quality management and clinical outcomes.

Finally, the generalized acceptance of a LATAM cardiac surgery board examination among the study respondents is a clear sign of the need for standardization of training in LATAM. This exam would not only assure the professional quality of the certifed surgeons but would also facilitate international mobility as well as increase the global acknowledgment and acceptance of LATAM cardiac surgeons. Collaborative work with more experienced associations is of utmost importance to achieve this goal.

### Limitations

The main limitation of this study is that the survey results might not necessarily represent the complete picture and reality of LATAM since 289 respondents are only a small fraction of the total cardiac surgery trainees and staf surgeons in LATAM. Moreover, other important topics/questions might still be missing and were not included in the survey to increase the survey completion rate since otherwise, the survey might be too long, and respondents would tend to abort the questionnaire. Finally, to avoid violating confdentiality and ensure the anonymity of the respondents, some results are limited to ranges rather than expressed as specific numeric values, thus reducing the specificity of some results.

## CONCLUSION

Cardiac surgery is considered a rewarding specialty among LATAM surgeons and trainees. However, significant improvements are required to reduce salary dissatisfaction, increase job and leadership opportunities, and narrow the gender and racial gap. In addition, the modernization and standardization of surgical training programs are also required to improve the training programs’ quality and the trainees’ satisfaction. All these improvements would reduce significant disparities in the specialty and an enhance regional response to the dynamic challenges in the field.

## Supplementary

### Supplementary Appendix Survey Questionnaire.

**Table:**

General questions (for both staff surgeons and residents)					
Gender	Male	Female			
Race	Caucasian	Black	Mestizo	Indigenous	Other:
Age					
Country					
City					
Institution	Private	Public			
Work hours per week	<40	40-80	80-120	> 120	
Position	Resident	Staff			
Do you have a leading position at your institution? What type of position?	Department head	Consultant	Chief resident	Leadership in any scientific society	
Is it easy to access to leading positions in cardiac surgery in your country?	Yes	No interest	No time	Not easy	Bad payment
Salary (USD)	< 1000 USD	1000-2500 USD	2500 - 5000 USD	5000-10.000 USD	> 10.000 USD
Are you satisfied with your salary?	Completely satisfied	Moderately satisfied	Unsatisfied		

USD=United States Dollar

### Questions for staff surgeons

**Table:**

Total length of residency (years)									
Country of residency									
Do you work in your country of origin?	Yes	No							
Did you have to train in general surgery prior to cardiac surgery?	Yes	No							
How long was the training in general surgery?	< 1 year	1 -2 years	2-4years	> 4 years					
Do you have critical care medicine training?	Yes	No							
How long was your critical care training?	3 months	3-6 months	6-12 months	1 -2 years	> 2 years				
How long was the training in cardiac surgery?	< 1 year	1 -2 years	2-4years	> 4 years					
Did you course any fellowship after your cardiac surgery training?	Congenital surgery	Aortic surgery	Minimally invasive surgery	Transcatheter interventions	Heart transplantation/heart failure	Other fellowship	No fellowship		
Did you have a salary during your residency?	Yes	No							
Did you have to pay for your cardiac surgery training?	Yes	How much (USD):	No						
Did you require any loans to pay your training?	Yes	No							
Years of experience as staff cardiac surgeon									
Number of cases per year	<50	50-100	100-150	> 150					
Number of cases per week	Total:	CABG	Mitral valve	Aortic valve	Aortic surgery	Congenital surgery	Heart transplantation	LVAD/ECMO	Other surgeries
Do you feel it is easy to find a new position as cardiac surgeon in your country?	Yes	No							

CABG=coronary artery bypass grafting; ECMO=extracorporeal membrane oxygenation; LVAD=left ventricular assist device; USD=United States Dollar

**Table:**

Do you have any relationship to university/ academic institutions?	Professor	Guest lecturer	Residence program director	Residents/students trainer		
Is research part of your duties?	Yes	No				
Do you have an academic title?	PhD	MSc	MBA			
Do you train residents/fellows?	Yes	No				
If you train residents, is it as part of an official residency program recognized by local governments, associations, universities, and academic/healthcare institutions?	Yes	No				
How many staff surgeons work in your department	1 surgeon	2 surgeons	2 - 5 surgeons	5 - 10 surgeons	> 10 surgeons	
Are all the staff surgeon positions occupied in your department?	Yes	No	1 surgeon missing	2 surgeons missing	3 surgeons missing	> 3 surgeons missing
Do you think that creating a standard cardiovascular exam, like the board exam in the United States of America or the European board exam, could be useful in Latin America to standardize the knowledge in our continent?	Yes	No				

### Questions for residents

**Table:**

Total length of residency (years)					
Length of the cardiac surgery training (*i.e.,* fellow or residency) in years					
Did you have to train in general surgery prior to cardiac surgery?	Yes	No			
How long was the general surgery training?	< 1 year	1 - 2 years	2 - 4years	> 4 years	
Do you have critical care medicine training?	Yes	No			
How long was your critical care training?	3 months	3 - 6 months	6 - 12 months	1 - 2 years	> 2 years
Do you have to pay for your cardiac surgery training?	Yes	How much (USD):		No	
Is your training/residency program an official residency program recognized by local governments, associations, universities, and academic/healthcare institutions, with an official title/diploma at the end of the training?	Yes	No			
Number of complete cases per week (own cases)					
Number of incomplete cases per week (essential parts of the operation)					

USD=United States Dollar

**Table:**

Total number of cases at the end of cardiac surgery training							
Number of night shifts per week							
How many residents are being currently trained in your department?	1 resident	2 residents	2 - 5 residents	5 - 10 residents	> 10 residents		
Are all the resident positions occupied in your department?	Yes	No	1 resident missing	2 residents missing	3 residents missing		
Are you satisfied with the quality of your residency/training?	Completely satisfied	Moderately satisfied	Unsatisfied				
When you finish your training, do you think good job opportunities as cardiac surgeon are available in your city/country?	Yes	No					
Are you willing to course a fellowship after you finish your training?	Congenital surgery	Aortic surgery	Minimally invasive surgery	Transcatheter interventions	Heart transplantation/heart failure	Other fellowship	No fellowship
Did you require any loans to pay your training?	Yes	No					
Do you think that creating a standard Cardiovascular exam, like the board exam in the United States of America or the European board exam, could be useful in Latin America to standardize the knowledge in our continent?	Yes	No					

**Table:**

How do you feel doing the following?			
Pacemaker implantation	Comfortable	Some insecurity	Uncomfortable
MRI reading (interpretation)	Comfortable	Some insecurity	Uncomfortable
CT-scan reading (interpretation)	Comfortable	Some insecurity	Uncomfortable
ECG reading (interpretation)	Comfortable	Some insecurity	Uncomfortable
Angiogram reading (interpretation)	Comfortable	Some insecurity	Uncomfortable
Shift on the ICU	Comfortable	Some insecurity	Uncomfortable
Transcatheter procedures	Comfortable	Some insecurity	Uncomfortable

CT=computed tomography; ECG=electrocardiogram; ICU=intensive care unit; MRI=magnetic resonance imaging
